# Exploring virulence in *Mycobacterium bovis:* clues from comparative genomics and perspectives for the future

**DOI:** 10.1186/s13620-023-00257-6

**Published:** 2023-09-28

**Authors:** Morgane Mitermite, Jose Maria Urtasun Elizari, Ruoyao Ma, Damien Farrell, Stephen V. Gordon

**Affiliations:** 1 UCD School of Veterinary Medicine, University College Dublin, Dublin, Ireland; 2 Faculty of Science, Vrije Universiteit Amsterdam, Amsterdam, Netherlands; 3 Department of Microbiology and Immunology, Weill Cornell Medical College, New York, NY 10065 USA; 4 UCD School of Medicine, University College Dublin, Dublin, Ireland; 5 UCD School of Biomolecular and Biomedical Science, University College Dublin, Dublin, Ireland; 6 UCD Conway Institute, University College Dublin, Dublin, Ireland

**Keywords:** *Mycobacterium bovis*, Tuberculosis, TB, Genomics, Virulence

## Abstract

Here we provide a summary of a plenary lecture delivered on *Mycobacterium bovis*, the bovine TB bacillus, at the *M. bovis* 2022 meeting held in Galway, Ireland, in June 2022. We focus on the analysis of genetic differences between *M. bovis* and the human pathogen *Mycobacterium tuberculosis* as a route to gain knowledge on what makes *M. bovis* function as an animal pathogen. We provide a brief historical background around *M. bovis* and comparative virulence experiments with *M. tuberculosis*, before moving to what we have learned from the studies of the *M. bovis* genome sequence. We discuss the need to translate knowledge on the molecular basis of virulence in *M. bovis* into improved control of bovine tuberculosis.

## Introduction

*Mycobacterium bovis* is a member of the *Mycobacterium tuberculosis* complex (MTBC), the group of related pathogens that causes tuberculosis (TB) in mammals [1]. A key feature of the MTBC pathogens is their adaptation to sustain in specific host populations: by this, we mean the ability of these pathogens to maintain cycles of infection, disease, and transmission within their preferred hosts. *Mycobacterium bovis* is seen as the archetypal animal-adapted member of the MTBC, able to sustain across cattle, badgers, deer, possums, etc. This contrasts with *Mycobacterium tuberculosis* which is the prototypic human TB pathogen but with apparently limited ability to sustain in domesticated animals or wildlife. The high degree of genetic identity between *M. bovis* and *M. tuberculosis* therefore offers the possibility of using comparative genomic approaches to identify the molecular basis of how *M. bovis* has evolved into such an effective, and problematic, animal pathogen. Through the identification of such pathogen adaptations, and understanding their mechanisms of action, we hope to better thwart *M. bovis* and ultimately accelerate eradication of bovine TB.

### Historical overview

It was Theobald Smith in 1896 who first described morphological, culture and pathological distinctions between TB bacilli isolated from humans and animals, leading to his suggestion of a distinct bovine TB bacillus [2, 3]. Through this work Smith showed how inoculation of cattle with bacilli isolated from either human sputum, or from cattle, led to distinct pathological outcomes in the infected cattle, with the bovine-derived bacilli showing greater virulence in cattle than human sputum-derived bacilli. The announcement in 1901 by Robert Koch at the 1901 British Congress on Tuberculosis that the bovine tubercle bacillus presented a negligible risk to humans, and that he did “*not deem it advisable to take any measures against it*” [4] sparked a major controversy. Multiple research studies were established that would subsequently show the risk of infection of the bovine bacillus to humans. A British Royal Commission established in 1901 to examine the evidence concluded in 1911 that humans “*must therefore be added to the list of animals notably susceptible to bovine tubercle bacilli*” [5]. While the bovine bacillus was often seen as a subtype of *M. tuberculosis* and hence referred to as *M. tuberculosis* var *bovis* or *M. tuberculosis* subsp *bovis*, the designation *Mycobacterium bovis* was established in 1970 [6].

It is worth returning to the point above relating to the early comparative virulence work on human and bovine bacilli. In addition to Smith’s work showing the attenuation of human-derived bacilli for cattle as compared to bovine varieties, von Behring also promoted the use of human bacilli as a vaccine against TB in cattle, termed ‘bovo-vaccine’. This was based on von Behring’s own work showing the attenuation of human bacilli for cattle [7, 8]. However, subsequent work was to show that inoculation of cattle with bovo-vaccine led to shedding of live human tubercle bacilli in milk [9], while others reported variable protection afforded by the vaccine [10], and the use of bovo-vaccine was ultimately abandoned. As described by Palmer and Waters [11] in their review of the US bovine TB eradication program, reports of variable virulence levels of human bacilli for cattle were often not clear cut. In his 1901 report as Chair of the Bureau of Animal Industry, Salmon [12] referred to work from Chauveau showing that “*human tubercular virus (sic) acts on the bovine species exactly like the tubercular virus which comes from the bovine species itself*”. In this same report Salmon also described work from Ravanel who fed 4 calves with human sputum, and that “*postmortem examination proved that all had become affected with tuberculosis, the lesions in two being quite extensive*.” Ravenel had also obtained bacilli from a child who died of TB meningitis; using this for infection studies he found that “*bacilli from this material have proved very virulent for bovine animals*” [12]. However, the exact designation of bacilli used in these early studies is often unclear, inoculation dose and routes varied, and immune status of the cattle used in the infection studies not stated.

To readdress virulence of *M. bovis* and *M. tuberculosis* in cattle, Whelan et al. [13] undertook experimental infections using *M. tuberculosis* H37Rv and *M. bovis* AF2122/97, which at the time were the best characterised genome sequenced isolates of the respective species. In their work, cattle infected with *M. tuberculosis* H37Rv and *M. bovis* AF2122/97 both gave positive skin test and interferon gamma tests, but cattle infected with *M. bovis* AF2122/97 showed extensive pathology while those infected with *M. tuberculosis* H37Rv showed no obvious pathological lesions. Of course, it could be argued that *M. tuberculosis* H37Rv is a laboratory-adapted strain; it was derived by Steenken and colleagues in the 1930s by in vitro passage of an *M. tuberculosis* strain termed ‘H37’ which itself had been isolated by Baldwin in 1905 [14]. Hence the apparent attenuation of *M. tuberculosis* H37Rv for cattle could be an artefact of its in vitro adaptation and not be representative of *M. tuberculosis* in general. To further explore the virulence of *M. tuberculosis* in experimental cattle infections, a strain of *M. tuberculosis* termed BTB1558, that had been isolated from the lesion of a Zebu bull in Ethiopia, was assessed in head-to-head comparisons with *M. bovis* AF2122/97 and *M. tuberculosis* H37Rv [15]. In this latter study, Villarreal-Ramos and colleagues found that *M. bovis* AF2122/97 generated more pathology in infected animals than either *M. tuberculosis* H37Rv or the *M. tuberculosis* BTB1558 strain. Hence both experimental infection studies have shown that the *M. tuberculosis* strains used showed reduced virulence as compared to *M. bovis* AF2122/97 in cattle.

Before we draw definitive conclusions from these experiments on the attenuation of *M. tuberculosis* in the bovine host per se, it should be noted that both *M. tuberculosis* strains used in the studies of Whelan et al. [13] and Villarreal-Ramos et al. [15] were from *M. tuberculosis* lineage 4, the so-called Euro-American lineage. There are nine currently defined lineages of *M. tuberculosis*, with many of these showing distinct geographical distributions [16, 17]. Reports of the isolation of *M. tuberculosis* from lineage 1 [18], 3 and 4 [19] from cattle in India suggests that conclusions derived from the study of *M. tuberculosis* BTB1558 and H37Rv may not apply across all *M. tuberculosis* lineages for cattle. Also, the immune status of an animal could modulate its susceptibility to infection and disease regardless of the genetic background of the infecting strain. Furthermore, virulence is not a ‘linear’ concept, and pathogen success is not necessarily defined by increased virulence for the host population to which the pathogen is adapted. Nevertheless, *M. tuberculosis* H37Rv and *M. bovis* AF2122/97 can provide ‘extremes’ in terms of the outcome of infection in cattle, with comparative analysis of these strains providing a route to define the molecular basis of *M. bovis* adaptation to cattle. Furthermore, the isolation of *Mycobacterium orygis* from cattle in South Asia [20–22] suggests that this MTBC species has also evolved a successful strategy to sustain in cattle populations [23], suggesting that comparative analysis using this species will also prove fruitful.

### *Mycobacterium *bovis genomics

The landmark publication of the genome sequence of *M. tuberculosis* H37Rv in 1998 [24] heralded a new era in our ability to explore the internal workings of mycobacterial pathogens. The *M. bovis* sequencing project came to fruition a few years later with the publication in 2003 of the complete genome sequence of *M. bovis* AF2122/97, a strain which had been isolated in Cornwall, UK in 1997 [25]. These genome sequences were elucidated using Sanger sequencing technology. The advent of ‘next generation’ sequencing approaches in the mid -2000s meant that mycobacterial pathogens can now be rapidly sequenced at a fraction of the cost of the original genome sequencing projects. The exquisite resolution and throughput provided by next generation sequencing has transformed the ability to trace the evolution of *M. bovis* [26] and track transmission across livestock and wildlife [27]. As other presentations at the *M. bovis* 2022 conference showcased the application of pathogen genome sequencing technology for epidemiological studies, we shall not deal with it in this review. Rather we will focus on what genomics has taught us about the functional capacity of the *M. bovis* genome.

As the first approach to the analysis of what makes *M. bovis* ‘tick’ as a successful animal pathogen, comparative analysis with the genome of *M. tuberculosis* strains, in particular *M. tuberculosis* H37Rv, was pursued [25]. This analysis revealed > 99% identity at the nucleotide level between the bacilli, as well as confirming the presence of several deletions from the *M. bovis* genome as compared to *M. tuberculosis* [25, 28]. Notably the RD4 locus was deleted in all isolates of *M. bovis* studied, hence identifying it as a useful target locus in developing simple PCR-based screens to differentiate *M. bovis* from other members of the MTBC [29, 30]. Another immediate finding of the genomic studies was that there were no unique genes per se in the genome of *M. bovis* compared to *M. tuberculosis* [25]. This latter finding indicated that the adaptation(s) of *M. bovis* to sustain in animal populations were not due to the presence of a particular gene that was lacking in human adapted bacilli. Instead, differential expression of genes across the human and bovine TB bacilli appeared a more likely basis for their unique host adaptations. Studies identifying differentially expressed genes and proteins between the animal- and human-adapted bacilli were therefore pursued as a route to defining their functional impact on host tropism.

An example of the impact of single nucleotide polymorphisms (SNPs) between the genomes of *M. bovis* and *M. tuberculosis* was the identification of a SNP in the *pykA* gene encoding pyruvate kinase, a key gene in the metabolism of carbohydrates that converts phosphoenolpyruvate to pyruvate which feeds into the TCA cycle. The *M. bovis* SNP leads to the inactivation of pyruvate kinase due to substitution of the glutamic acid residue at position 220, located at the enzyme’s active site, with aspartic acid [31]. The lack of a functional pyruvate kinase means *M. bovis* cannot use carbohydrates as a sole carbon source, an insight that provided an explanation for the long-held observation that *M. bovis* is unable to grow on glycerol as a sole carbon source. Studies have shown that inactivation of the *pykA* of *M. tuberculosis* resulted in catabolism of fatty acids for energy production, whereas complementation of *M. bovis* with a functional *pykA* led to carbon conservation through gluconeogenesis and the glyoxylate cycle [32]. Whether the inactivation of the *M. bovis pykA* is deleterious for the bacillus, or offers some advantage via metabolic remodelling, is unknown. Interestingly, analysis of an expanding *M. bovis* clone in the UK (termed ‘type 17’) revealed a deletion that altered carbon metabolism such that propionate derived from fatty acid metabolism was shuttled to generate pyruvate, hence perhaps compensating for the inactivation of PykA [33].

With the advent of ‘Next Generation’ sequencing technologies *M. bovis* AF2122/97 was re-sequenced using both Illumina and PacBio approaches [34]. A surprising finding from this resequencing was the presence of an approximately 3.1 kb region of DNA which had been missed in the original *M. bovis* AF2122/97 sequencing project. This 3.1 kb region corresponded to the RD900 locus which had been described by Bentley and colleagues as present in *Mycobacterium africanum* but deleted from many other members of the MTBC, including *M. bovis* [35]. However, the resequencing and de novo assembly showed that this locus was in fact present in the genome of *M. bovis* AF2122/97. RD900 contains genes encoding a potential transporter and two protein kinases (PknH1 and PknH2); these latter proteins are through to transduce extracellular signals and trigger signalling cascades. The genome of *M. tuberculosis* H37Rv lacks the RD900 locus and has a single PknH [24]. Subsequent work by Mata and colleagues [36] showed that expression of the *M. tuberculosis* PknH orthologue in *M. bovis* led to altered expression of 154 genes in *M. bovis*, indicating that the *M. tuberculosis* and *M. bovis* PknH protein kinases have distinct functional activities.

To update the functional annotation of the *M. bovis* genome, Malone et al. [37] used a combination of quantitative transcriptomics and proteomics to identify the most highly expressed genes and most abundant proteins in *M. bovis* AF2122/97 and compared this to parallel datasets from *M. tuberculosis* H37Rv. This revealed 77 genes upregulated in *M. bovis* AF2122/97 at both the RNA and protein level as compared to *M. tuberculosis* H37Rv [37]. To explore the mechanistic basis for differential gene expression analysis across the two species, a transcription factor enrichment analysis revealed significant association of a core set of transcription factors with the differentially expressed genes. Among these transcription factors was SigK and PhoPR which we discuss in greater detail below.

A further update to the *M. bovis* AF2122/97 genome annotation was performed by Farrell and colleagues [38]. Their analysis focused on genes that had originally been described as ‘hypothetical’, or of unknown function. These latter designations represented a limitation of the initial genome annotation, namely a reliance on ascribing potential gene function based on information available from other organisms. Due to the paucity of comparative data available at the original time of genome annotation in the early 2000s, almost 30% of coding sequences in a genome would have no known function and comparison to other organisms would reveal limited information. However, with the exponential increase in bacterial genomes sequenced and analysed using ‘omic methodologies over the past 20 years, plus the advent of more sophisticated computational tools for comparative analysis of orthologous genes, many *M. bovis* hypothetical proteins could now be placed into potential functional groupings. Through this analysis, Farrell et al. [38] updated functional information for over 600 M*. bovis* genes and updated 69 gene names. The current *M. bovis* AF2122/97 genome and functional annotation is available via the DDBJ/ENA/GenBank accession number LT708304.

As highlighted above, the transcriptomic and proteomic information layered onto the genome sequence identified key differentially regulated loci between *M. bovis* AF2122/97 and *M. tuberculosis* H37Rv. Given the divergent interaction of these species with cattle, the genes and proteins identified as differentially expressed provide points a focus for research efforts that seek to refine our understanding of *M. bovis* virulence and host adaptation. In the following sections we focus on regions that show differential regulation in *M. bovis* and summarise what ongoing studies are revealing about the potential functional impacts of these differences.

### The SigK regulon

**Fig. 1:**
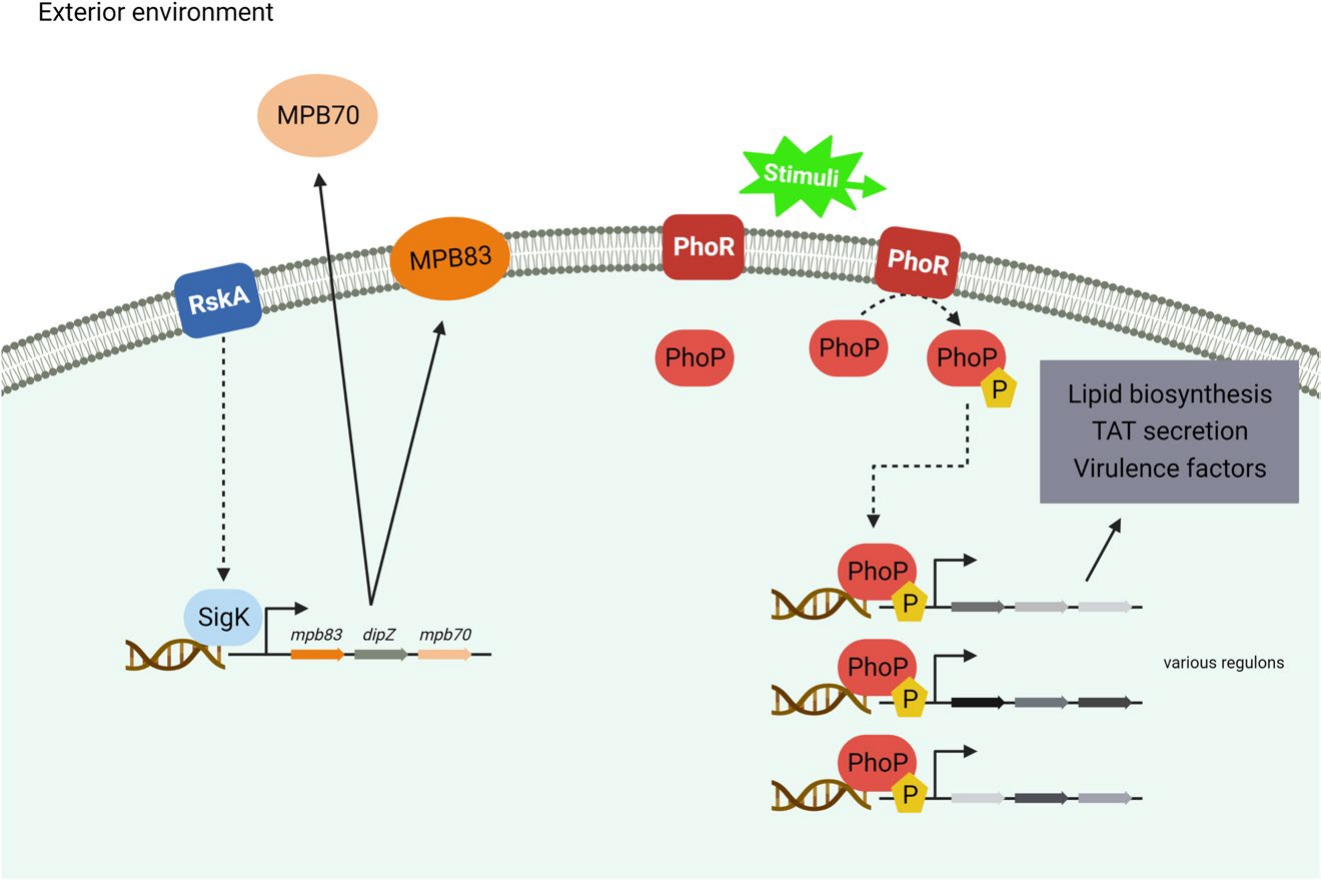
Overview of the SigK and PhoPR systems in *M. bovis*. The SigK system is shown on the left-hand side of the figure. The regulator of the SigK sigma factor, RskA, is mutated in *M. bovis* leading to constitutive expression of the regulon which includes the *mpb83-dipZ-mpb70* locus. MPB83 is a glycosylated lipoprotein and cell wall associated, while MPB70 is secreted. On the right-hand side of the figure the PhoPR system is shown. PhoR senses environmental stimuli that trigger phosphorylation of the PhoP transcriptional regulator; phosphorylated PhoP in turn controls expression of genes involved in diverse functions, such as secretion of virulence effectors and cell wall glycolipid biosynthesis

SigK is an extra cytoplasmic function (ECF) sigma factor which, as the name suggests, regulates responses to extracellular conditions. Behr and colleagues were the first to show that the SigK regulon was dysregulated between *M. bovis* and *M. tuberculosis* [39, 40]. They showed that the anti-sigma factor regulator of SigK, RskA, harboured a mutation in *M. bovis* that inactivated its function, leading to constitutive expression of the SigK regulon in *M. bovis* [39, 41]. Amongst the genes under the control of SigK are *mpb70* and *mpb83*, encoding the proteins MPB70 and MPB83, respectively (Fig. 1). The ‘MPB’ nomenclature was introduced by Nagai et al. [42] with, for example, MPB70 standing for “*a mycobacterin which is a protein fraction from M. bovis BCG as a band with a relative mobility of 0.70*”. While MPB70 is abundant in culture filtrates of *M. bovis* it was not detected within sonicated extracts from the bacteria prewashed with aqueous buffers, indicating efficient secretion of the protein [43]. In contrast, MPB83 was detected in sonicated lysates of washed bacilli, and flow cytometry of *M. bovis* BCG Tokyo with a monoclonal antibody against MPB83 showed that MPB83 was anchored at the bacterial surface [44].

It is noteworthy that upregulated expression of the SigK regulon was also seen in *M. orygis*, but by an independent mutation [40]. This is intriguing because *M. orygis* has been suggested to be another bovine-adapted member of the MTBC [20, 21, 23], implying a potentially selective advantage to upregulation of the SigK regulon in pathogens that sustain in bovine hosts. Furthermore, it was demonstrated [41] that RskA is not a simple negative regulator that sequesters its associated sigma factor SigK and releases it upon environmental stimulation. Instead, RskA possesses a dual function of activator-inhibitor of SigK. Both the *M. bovis* and *M. orygis* mutated forms of RskA do not result in increased SigK activity simply due to loss of the RskA inhibitory function, but rather by enhancement of the RskA activator function, hence leading to increased SigK activity [41].

The genes under the control of SigK are restricted to two loci on the genome [39, 40, 45]. One region is the *mpb70/83* locus that encodes: MPB83, a homolog of MPB70 that is anchored to the membrane and post-translationally modified by acylation and glycosylation; DipZ, an integral membrane protein; MPB70, a secreted protein; and Mb2901, a possible transmembrane protein. The second region is the SigK locus itself, which comprises: Mb0457c and Mb0456c, proteins of unknown function; UfaA1, a phospholipid synthase; Mb0453c, again of unknown function; and finally, SigK and RskA [45]. While ECF sigma factors are known to control relatively small regulons, the SigK regulon is remarkably small; in comparison to the 10 genes encompassed by the SigK regulon, SigH regulates the expression of 41 genes [46], while SigC controls the expression of 18 genes [47].

### MPB70

The best characterised member of the SigK regulon is MPB70. The structure of mature MPB70 was determined by nuclear magnetic resonance (NMR) spectroscopy in 2003 [48], revealing that MPB70 folds into a globular tertiary structure made of a seven stranded β-barrel adjoined to eight α-helices that pack on one side of the barrel. This study unveiled significant similarity between the backbone topology of residues 27 to 159 of MPB70 (i.e., 133 amino acids of the total 163 amino acid sequence of MPB70) and the tertiary structures of the third and fourth FAS1 (Fasciclin-like) domains of the *Drosophila melanogaster* Fasciclin I protein. This latter protein was the first for which the structure of a FAS1 domain was determined, using X-ray crystallography, and therefore provided the structural prototype for the FAS1 domain [49]. In general, FAS1 domains are made of approximately 140 amino acids and are present in extracellular proteins, either secreted or bound to the plasma membrane, where they are implicated in cell-to-cell, cell-to-extracellular matrix, and extracellular matrix structure adhesion [50], or in homophilic domain-to-domain trans-interactions between FAS1 proteins to mediate cell-to-cell interactions [51]. The FAS1 domain is an ancient structural motif, as it is shown to be extensively represented in animal, bacteria, and plant proteins [52, 53]. FAS1 proteins were found in both eubacteria and archaea, suggesting that the domain originated prior to the last universal common ancestor, making it an ancient adhesive extracellular domain [54]. While FAS1 proteins are widely represented throughout various—if not all—biological groups, they are not ubiquitous (especially in microorganisms), suggesting that the FAS1 domain takes part in specialized cellular interactions [50].

A range of human proteins contain FAS1 domains, including periostin, TGFBI/βig-h3, stabilin-1 and stabilin-2. The latter two stabilin proteins are homologous transmembrane proteins with C-terminal cytosolic tails [55]. The stabilins contain a total of seven FAS1 domains, composed of three dual FAS1 domain tandems and one isolated FAS1 domain, each separated by a varying number of Epidermal Growth Factor (EGF) repeats and an X-link domain, with these latter domains thought to be involved in phosphatidylserine and hyaluronic acid binding, respectively [50, 55]. Both stabilin-1 and stabilin-2 are expressed in various cell types, including tissue macrophages. Recombinant mammalian cell lines that had been engineered to express high levels of stabilin-2 were shown to become sticky and aggregate, with the FAS1 domains of stabilin-2 found to be responsible for homophilic cell–cell adhesion [51]. This work also revealed an intriguing characteristic of MPB70. Briefly, Park et al. [51] performed a series of aggregation assays with a mouse fibroblast cell line expressing (or not) stabilin-2 and firstly demonstrated that stabilin-2 mediated cell aggregation in suspension. Then, to find out if the FAS1 domains of stabilin-2 in particular were responsible for cell aggregation, the authors used recombinant MPB70 – used as a model FAS1 protein carrying a unique FAS1 domain—as a further element in their aggregation assay. The authors expected that the FAS1 domains of MPB70 would inhibit cell aggregation by interfering with homophilic interactions of stabilin-2. However, the opposite happened: when MPB70 was present in the media it further enhanced the aggregation of the cells. This suggested a role of MPB70 in cellular aggregation.

### MPB70: host interactions

Queval et al. [56] explored the interaction of *M. bovis* and *M. tuberculosis* with human and bovine macrophages. They observed that the in vitro infection of bovine blood monocyte-derived macrophages (MDMs) with *M. bovis*, but not *M. tuberculosis*, promoted the formation of multinucleated cells (MNC) by membrane fusion. Furthermore, they only obtained the multinucleation phenotype when using bovine MDMs; infection of human MDMs with *M. bovis* did not trigger multinucleation, suggesting a species-specific interaction. Multinucleation is a complex and multi-step process which, amongst other determinants, requires the interaction and fusion of cellular membranes. As seen previously, stabilin-2 facilitated cellular membrane interaction leading to aggregation and played a key role during myoblast multinucleation [51, 57, 58]. The observation by Park and colleagues that MPB70 enhanced cell aggregation led to the hypothesis that the differential phenotype of multinucleation of bovine MDMs could be a consequence of the differential profile of expression of MPB70 by the two mycobacterial strains (as *M. bovis* has constitutive expression of MPB70, while MPB70 expression is induced in *M. tuberculosis* after phagocytosis).

To address this hypothesis, Queval et al. [56] assessed whether a lack of MPB70 in *M. bovis* would prevent the macrophage multinucleation phenotype they had seen. An in vitro infection of bovine MDMs was therefore performed with using an *M. bovis* AF2122/97 wild type strain, an *M. bovis* AF2122/97 Δ*mpb70* knockout mutant, and the complemented *M. bovis* AF2122/97 Δ*mpb70* knockout with synthesis of MPB70 restored. At 24 h post infection lower numbers of MNC were obtained after infection of bovine MDMs with the Δ*mpb70* knockout strain, similar to numbers obtained in the non-infected cells. They furthermore demonstrated that infection of bovine macrophages with the complemented *M. bovis mpb70* knockout strain led to similar MNC levels to those observed after infection with *M. bovis* wild type. These results therefore supported the hypothesis that MPB70 played a role in the phenotype of multinucleation of bovine MDMs.

The observation of *M. bovis*-specific interaction with bovine immune cells, dependent on MPB70, are intriguing. However, many questions remain. For example, Queval et al. [56] studied cellular phenotypes over 24 h; it is possible that the MPB70 effect may be temporal, specific to early time points in vitro, and that later time points may reveal multinucleation for both species. At the cellular level, we do not know what the macrophage proteins are that interact with MPB70. While stabilin-2 or other FAS-1 containing proteins may be candidates, this needs to be clarified. The in vitro observation of multinucleation was made with bovine blood MDMs; however, in vivo, tissue-resident alveolar macrophages are of a different origin and are functionally distinct to recruited, interstitial macrophages. It would therefore be interesting to see what, if any, differential phenotype would be observed in bovine alveolar macrophages infected with the *M. bovis* wild type, the *mpb70* knockout mutant, and complemented *mpb70* mutant. Experimental infection of cattle with the *M. bovis* AF2122/97 Δ*mpb70* knockout would also reveal how the phenotypes observed in vitro translate to the in vivo situation. Indeed, it remains to be established if MPB70 is a lone player in host interactions, or if other genes in the SigK regulon also play a role.

### PhoPR

Another system that showed differential expression between *M. bovis* and *M. tuberculosis* was the PhoPR system (Fig. 1). PhoPR is a master regulator of virulence in *M. tuberculosis*. It is a two-component system, made up of a sensor kinase (PhoR) that detects changes in environmental stimuli, and a response regulator (PhoP). When activated by PhoR, PhoP forms a dimeric structure via its N-terminal region, and its C-terminal binding domain directly binds target sequences across more than 30 M*. tuberculosis* genes to regulate their expression [59, 60].

A major virulence system that is controlled by PhoPR is the ESX-1 secretion system which exports, among other factors, the dominant T-cell antigens ESAT-6 (EsxA) and CFP-10 (EsxB), and the ESX-1 secretion-associated proteins (Esp) EspA and EspC [61]. The latter proteins are encoded in an operon, *espACD*, which is located distally from the main ESX-1 locus. Secretion from the ESX-1 locus is co-dependent on both the ESX-1 and *espACD* loci, with PhoP binding upstream of *espACD* to regulate ESX-1 secretion [61]. The PhoPR system also regulates the synthesis of many lipids restricted to pathogenic members of the MTBC such as diacyltrehaloses (DAT), polyacyltrehaloses (PAT), and sulfolipids (SL) [62, 63]. The major binding site of the PhoP regulator is the small non-coding RNA (ncRNA) encoded by *mcr7* that modulates the activity of the Twin Arginine Translocation (TAT) secretion apparatus. In PhoP mutants *mcr7* expression is abolished, which in turn affects the secretion of TAT substrates, such as the Ag85 complex [60]. The PhoPR system is hence a critical system for the control of a range of virulence functions in *M. tuberculosis*.

Given the central role of PhoPR in controlling virulence gene expression, it was a surprise when Gonzalo-Asensio et al. [64] reported defects in the PhoPR system in *M. bovis*. Specifically, they showed that *M. bovis* PhoR harbours a mutation at codon 71 that replaces a glycine with an isoleucine (Gly71Ile), leading to a defective PhoPR system. The apparent loss of a functional PhoPR in *M. bovis* compared to *M. tuberculosis* explains some of the known differences across the bacilli; for example, the absence of sulfolipid in *M. bovis* compared to *M. tuberculosis*. However, if the PhoPR system controls the secretion of ESAT-6 and CFP-10, antigens that are known to be secreted by *M. bovis*, how does the bovine pathogen still secrete these proteins? To explain this Gonzalo-Asensio and colleagues showed that deletion of the RD8 region from *M. bovis* removed *espACD* upstream control regions which, in combination with SNPs in the *espACD* promoter, allow *espACD* expression and hence ESX-1 secretion [64]. This again shows how study of genetic differences between *M. bovis* and *M. tuberculosis* can reveal the molecular basis for functional differences between the bacilli.

While the PhoPR system shows differences between *M. bovis* and *M. tuberculosis*, it is not the case that PhoPR is non-functional in *M. bovis*. Garcia et al. [65] showed that inactivation of PhoP in *M. bovis* led to decreased ability of the mutant to block phagosome maturation after engulfment by macrophages. Through microarray analysis of global gene expression, they also showed that the *M. bovis phoP* mutant had altered expression of 70 genes as compared to the *M. bovis* wild type, including genes involved in sulfolipid biosynthesis and transport, oxidative stress responses, and ESX-1 secretion. The *M. bovis phoP* mutant was also more sensitive to acid stress than the wild type, a stress condition that mimics the in vivo environment. Subsequent work by Garcia et al. [66] revealed that PhoP controls the production and secretion of ammonia from *M. bovis* that buffers against acid stress. Our own work (Urtasun-Elizari and Gordon, unpublished) has also found that inactivation of the *phoPR* locus in *M. bovis* leads to altered expression of multiple genes, again indicating that the locus is functional in *M. bovis*. However, the *M. bovis* PhoR orthologue may respond to distinct signals to that of *M. tuberculosis*.

### Future perspectives

An organism’s genome is often referred to as its ‘genetic blueprint’, as if it were a detailed plan to build the organism from scratch. However, the analogy is flawed; the genome is better viewed as a parts list, and a partial one at that, with no instructions on how the various parts come together to build a functioning organism. Through analysis of the *M. bovis* genome we aim to translate the genome information into an understanding of what makes the pathogen ‘tick’ in terms of its ability to infect and transmit across animal populations. As described herein, insights gleaned to date include an understanding of how evolution has shaped the genome of *M. bovis* to be distinct from that of the human-adapted pathogen *M. tuberculosis*, altering the expression of multiple genes that are involved in host–pathogen interaction. These findings have provided targets for us to focus our efforts on elucidating the mechanistic basis of pathogenesis. Such ‘omics studies parallel the use of genomics to define *M. bovis* population structure and transmission dynamics, to identify novel antigens, and to develop rapid nucleic acid diagnostics. Genomics is now a cornerstone of *M. bovis* research. We look forward to the next international *M. bovis* meeting to see genomics research translated to improved bovine TB control.

## Data Availability

Not applicable.

## Abbreviations

MTBC: *Mycobacterium tuberculosis* Complex
SNP: Single nucleotide polymorphism
FAS1: Fasciclin-like domain
MNC: Multinucleated cell
